# LINC317.5 as a novel biomarker for hypertriglyceridemia in abnormal glucose metabolism

**DOI:** 10.1038/s41420-024-01968-7

**Published:** 2024-04-26

**Authors:** Yixue Yang, Mengzi Sun, Shoumeng Yan, Nan Yao, Xiaotong Li, Caihong Wu, Zibo Wu, Fengdan Wang, Weiwei Cui, Bo Li

**Affiliations:** 1 Department of Epidemiology and Biostatistics, School of Public Health, Jilin University, Changchun, 130021 P. R. China; 2 The First Affiliated Hospital of Xi’an Jiaotong University, International Obesity and Metabolic Disease Research Center, Xi’an, 710061 P. R. China; 3 Global Health Institute, Xi’an Jiaotong University, Xi’an, 710115 P. R. China; 4 School of Nursing, Jilin University, Changchun, 130021 P. R. China; 5 Department of Nutrition and Food Hygiene, School of Public Health, Jilin University, Changchun, 130021 P. R. China

**Keywords:** Gene regulation, Long non-coding RNAs, Dyslipidaemias

## Abstract

The global rise in prediabetes and diabetes, with type 2 diabetes (T2DM) being predominant, highlights the association between T2DM and hypertriglyceridemia (HTG). Patients with both abnormal glucose levels and HTG require increased attention due to higher risks of complications and mortality. Therefore, this study aimed to find the key long non-coding RNA (lncRNA) of HTG in the abnormal glucose metabolism patients. We collected blood samples for RNA sequencing experiments and blood samples for validation in population. We have conducted RNA sequencing, weighted gene co-expression network analysis (WGCNA), quantitative real‐time polymerase chain reaction (qRT-PCR) in a 82-vs-82-sample-size population and insulin induced HepG2, RNA- Fluorescence in situ hybridization (FISH) and Cell Counting Kit-8 (CCK-8). We also explored lipid metabolism related transcription factor and the related protein expression and processed key lncRNA by both interference expression and overexpression, and the related consequences were rescued by its target mRNA. ENST00000540317.5 (LINC317.5) was lower in HTG with abnormal glucose metabolism and was found in both cytoplasm and nucleus in HepG2, inversely regulating the accumulation of TG and its target mRNA *TKFC*. Relative expression of peroxisome proliferator-activated receptor alpha (*PPARα)* and peroxisome proliferator-activated receptor gamma (*PPARγ)* were decreasing, and *SREBP-1c* (sterol regulatory element-binding protein-1c) was increasing of the interference expression of LINC317.5. Interference expression of LINC317.5 significantly decreased the protein expression of *ACADM* and *CPT1A*, whereas increased the protein expression of *FAS* and *ACC1*. *TKFC* partly reduced the triglyceride (TG) accumulation of LINC317.5. In conclusion, we suggested LINC317.5-*TKFC* as a key for TG accumulation in the HepG2-insulin resistant (IR). These might provide information of non-invasive biomarkers for the HTG with abnormal glucose.

## Introduction

In the worldwide, more than 470 million people were believed to have prediabetes by 2030 [1], and 693 million diabetes patients were expected by 2045 [2]. Type 2 diabetes mellitus (T2DM) accounts for about 90%–95% of patients with diabetes. It was reported that among T2DM patients, the prevalence of hypertriglyceridemia (HTG) might be 50% [3]. Triglyceride (TG) level was closely related to insulin resistance-compensated hyperinsulinemia instead of simply increasing with the increase of hyperglycemia [4]. Furthermore, T2DM patients with HTG were more likely to have diabetic complications [5], while diabetic patients with higher serum TG level were likely to have higher mortality rate [6]. Therefore, no matter from a public health perspective or a clinical perspective, patients with both abnormal glucose and HTG should be paid more attention.

A kind of transcripts longer than 200 nucleotides which could bind DNA, RNA or protein was called Long noncoding RNA (lncRNA) [7], and lncRNAs were also emerging being found to have regulatory effects on lipid metabolism [8]. lncRNAs might be involved in the entire biological process of prediabetes [9]. TCONS_00334653 and PVT1 were proved to be the key lncRNAs and potential therapeutic or diagnostic targets for prediabetes along with HTG [10]. Currently, researchers are still searching for novel reliable non-invasive biomarkers instead of the invasive sampling methods for the diagnostic protocols [11, 12]. Conventional methods usually described the correlation structure between thousands of genes and a sample trait [13], but weighted gene co-expression network analysis (WGCNA) could solve the problem.

Besides, mRNA played an important role in the study of HTG as well. By querying the KEGG database, it can be observed that many mRNA genes were involved in the glycerolipid metabolism pathway, such as triokinase and FMN cyclase (*TKFC*) (annotated as 2.7.1.29 in the pathway diagram) [14].

This study aimed to identify the key lncRNA of HTG in the abnormal glucose metabolism patients. Then, validation the key lncRNA in a larger sample size of the population and vitro experiments using insulin induced the HepG2. The effect of the key lncRNA on TG was verified by detecting changes in the expression levels of transcription factors and related enzymes of lipid metabolism in (insulin resistant-HepG2) HepG2-IR. Our results might provide the information of non-invasive biomarkers for the HTG with abnormal glucose.

## Results

### Identification of key lncRNA by WGCNA and validation

**Fig. 1:**
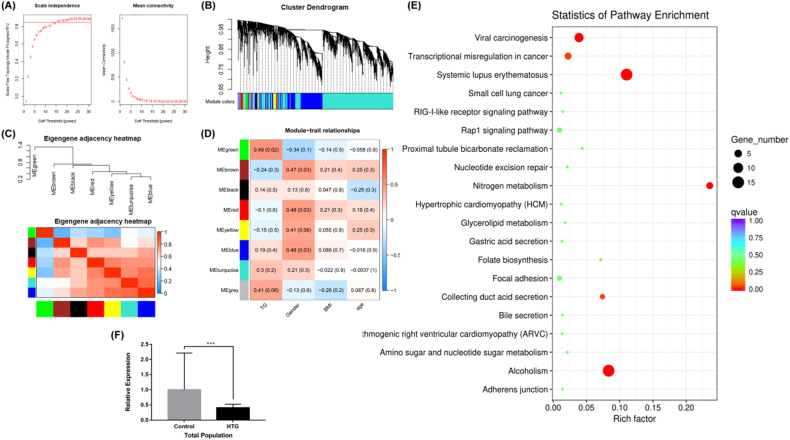
Identification of key lncRNA of HTG in the abnormal glucose metabolism by WGCNA. **A** Analysis of the scale-free fit index for various soft-thresholding powers. The left chart showed the correlation coefficients of log(k) and log(p(k)) corresponding to different soft thresholds; the right chart showed the mean values of gene adjacency coefficients corresponding to different soft thresholds, reflecting the average connectivity level of the network. **B** Hierarchical clustering tree and co-expression module of lncRNAs. At the top of the graph was a clustering tree of lncRNAs, and at the bottom were different modules cut from the dynamic cutting tree (different colors represent different modules). **C** The heat map plot of the adjacencies of modules (Red represented high adjacency (positive correlation), while blue color represented low adjacency (negative correlation)). **D** Heatmap of the module–trait relationships. It was represented the Pearson correlation coefficients and *P*-values of the correlation. Each row corresponded to a module gene, column to a trait. The cells were color coded by correlation according to the color legend. **E** Top 20 KEGG pathways of lncRNAs in abnormal glucose metabolism with HTG. The *X*-axis shows the rich factor and the Y-axis showed the KEGG terms. **F** The relative expression and its standard deviation of LINC317.5 in the abnormal glucose metabolism (with HTG and NTG) (The relative expression was calculated based on the target gene expression levels in the control group. ***P < 0.001) (HTG: hypertriglyceridemia; NTG normal triglyceride).

We selected lncRNAs in the top 5000 of MAD in 24 participants for subsequent analysis. As shown in Fig. 1A, the scale-free topology index was 0.85 when the soft-threshold power was defined as 16, which the network conformed to the power-law distribution and closer to the real biological network state. The dynamic hierarchical tree cutting algorithm was used to detect co-expression module according to the weight of lncRNAs, and the results of modules were shown as Fig. 1B. We have merged the modules with the number of lncRNAs was less than 30 and the height of the merged module was set to 0.25. Finally, black, blue, brown, green, gray, red, turquoise, and yellow (different colors represent different modules) 8 modules were obtained. Figure 1C was the heat map plot of the adjacencies of modules which represented the correlation between different modules. The most representative gene set in each module represented the overall level of gene expression in the module as the first principal component of the module eigengene (ME). Two modules corresponding to the sample trait were finally extracted for further functional enrichment analysis, and it was the green module had the significant correlation with TG in 24 samples in this study (correlation coefficient = 0.49, *P* = 0.02) (Fig. 1D). Then, we put the lncRNAs of green module into KEGG pathway analysis for further elucidation of the functional properties via co-relation method, as shown in Fig. 1E. We selected the glycerolipid metabolism pathway, which was the aim of our study, for subsequent analysis. Only one target mRNA was enriched in this pathway, it was *TKFC*. The *TKFC* was also enriched in other pathways, including RIG-I-like receptor signaling pathway, Carbon metabolism pathway, and Metabolic pathways. There were 7 lncRNAs including ENST00000533393.1, ENST00000543162.2, ENST00000540317.5, ENST00000451389.7, ENST00000537594.1, TCONS_00059583, and ENST00000535152.1 of *TKFC* in the RNA sequencing database.

The expression patterns of the 7 lncRNAs were verified by the GSE130991 data set, which involved 34 abnormal glucose metabolism patients with HTG and 64 abnormal glucose metabolism patients with NTG. RNA sequencing is superior to microarray for characterizing transcriptomes, however, data for GSE130991 were obtained by GPL20265 (HTA-2_0) Affymetrix Human Transcriptome Array 2.0. The probes in the data set are early and not enough to detect all genes. Only lncRNA- LINC317.5 (ENST00000540317.5) was founded in GSE130991; therefore, we selected this lncRNA to validate by the qRT-PCR. As shown in Fig. 1F, there was significant difference in 82 abnormal glucose metabolism patients with HTG and 82 patients with NTG by qRT-PCR (relative expression was 0.41, *t* = -4.404, *P* < 0.001).

The ROC curves for the relative expressions of LINC317.5 (AUC = 0.696, 95%CI 0.614-0.778, *P* < 0.001) in the abnormal glucose metabolism with HTG. Thus, we selected the LINC317.5 as the potential biomarker of HTG in the abnormal glucose metabolism patients for further exploration.

### The location of LINC317.5 in the HepG2 and the setting-up of HepG2-IR

**Fig. 2:**
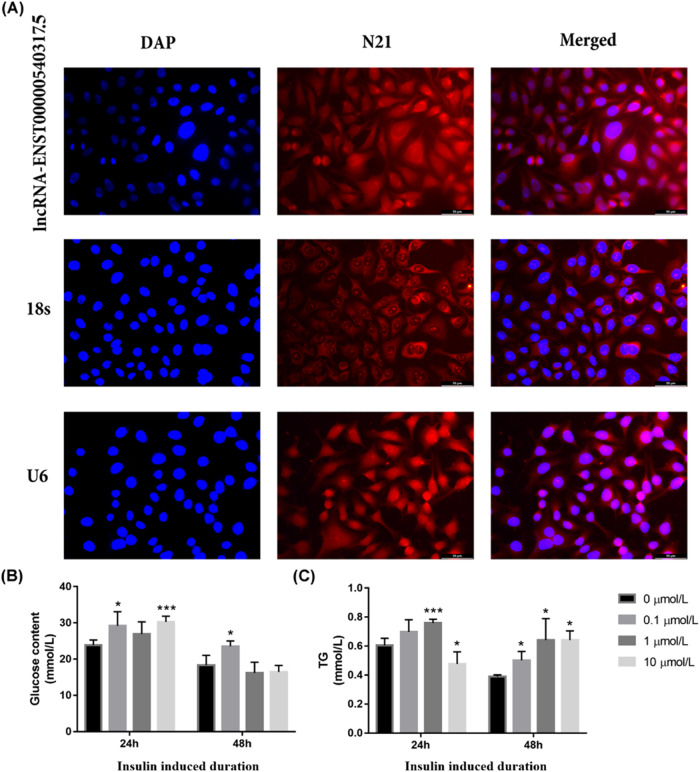
FISH localization of LINC317.5 in HepG2 cell and setting-up of HepG2-IR models. **A** RNA-FISH images showing that LINC317.5 was predominantly localized in both cytoplasm and nuclei. 18 s was mainly localized in the cytoplasm and U6 was mainly localized in the nuclei, they were used as a positive control. **B** The standardized extracellular glucose content for HepG2-IR model set up by different concentration of insulin and different induced duration (**C**) The TG level for HepG2-IR model set up by different concentration of insulin and different induced duration (*P < 0.05, **P < 0.01, ***P < 0.001) (DAP and N21: two specific dyes from the FISH kit; TG: triglyceride).

To investigate the mechanism of LINC317.5 during TG increasing, subcellular localization of LINC317.5 using FISH was performed. The results showed that LINC317.5 was found in both cytoplasm and nucleus in HepG2 (Fig. 2A).

Then, the HepG2-IR was constructed by different concentration insulin induced, and tested by extracellular glucose content and TG content. When successfully establishing the HEPG2-insulin resistance model, compared to the control group, the glucose and TG concentration in the cell environment may change. Owing to the decrease of the cell’s response to insulin, the ability of using glucose of cells will reduce. This may result in an increase in the glucose concentration in the cell environment. Meanwhile insulin resistance often typically leads to an imbalance in fatty acid synthesis and oxidation, resulting in increased synthesis and decreased breakdown of TG. Therefore, in the insulin resistance model, the TG concentration in the cell environment may increase. Only after treating the cells with 0.1 µmol/L of insulin for 48 h, there were significant increases in both TG concentration and glucose concentration in the cell culture environment compared to the control group. Therefore, 0.1 µmol/L insulin induced for 48 h was identified as the modeling condition (Fig. 2B, C).

### Effects of LINC317.5 on TG accumulation

**Fig. 3:**
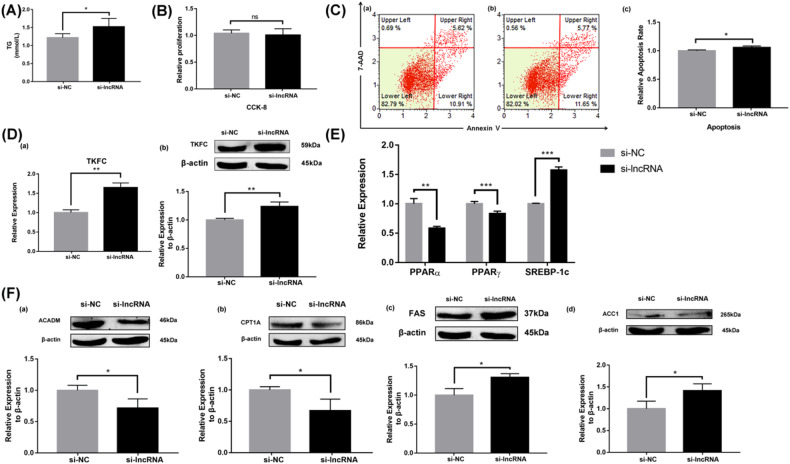
Knockdown of LINC317.5 regulates the lipid metabolism-related gene, transcription factor, and protein expressions in the HepG2-IR model. **A** The TG level of si-lncRNA and si-NC in the HepG2-IR model. **B** The effect of knockdown of LINC317.5 on the activity of HepG2-IR models with CCK-8. **C** The effect of knockdown of LINC317.5 on apoptosis of HepG2-IR model with flow cytometry (**a**) si-NC; (**b**) si-lncRNA; (**c**) Relative apoptosis rate. **D** The *TKFC* relative expression after knockdown of LINC317.5 in the HepG2-IR model; (**a**) for gene expression by qRT-PCR and (**b**) for protein expression by western blot. **E** The effect of knockdown of LINC317.5 on transcription factors of HepG2-IR model with qRT-PCR. The housekeeping gene which was used to establish the relative expression of the analyzed genes was β-actin. **F** The lipid metabolism-related protein expression of knockdown of LINC317.5 in the HepG2-IR (ACADM, CPT1A, FAS, ACC1). Western blot experiments have been repeated three times (The relative expression was calculated based on the target gene expression levels in the si-NC group. The relative proliferation was calculated based on the proliferation level in the si-NC group. The relative apoptotic rate was calculated based on the apoptotic rate in the si-NC group. *P < 0.05, **P < 0.01, ***P < 0.001) (TG: triglyceride).

**Fig. 4:**
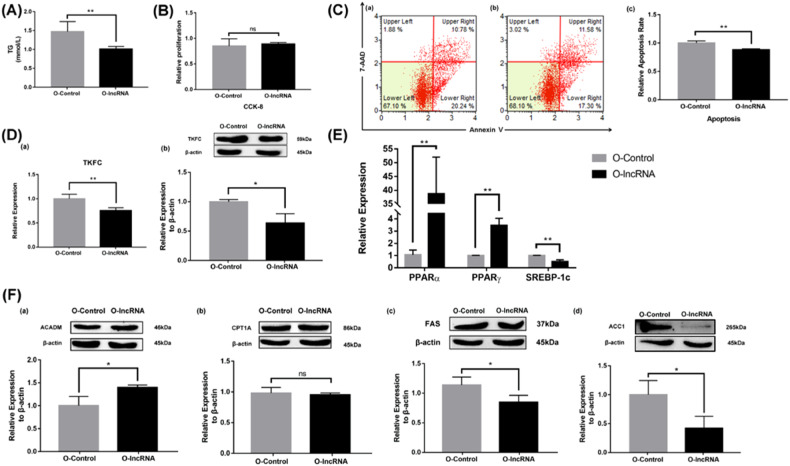
Overexpression the LINC317.5 regulates the lipid metabolism-related gene, transcription factor, and protein expressions in the HepG2-IR model. **A** The TG level of O-lncRNA and O-Control in the HepG2-IR model. **B** The effect of overexpression the LINC317.5 on the activity of HepG2-IR models with CCK-8. **C** The effect of overexpression the LINC317.5 on apoptosis of HepG2-IR model with flow cytometry (**a**) O-Control; (**b**) O-lncRNA; (**c**) Relative apoptosis rate. **D** The *TKFC* relative expression after overexpression the LINC317.5 in the HepG2-IR model; (**a**) for gene expression by qRT-PCR and (**b**) for protein expression by western blot. **E** The effect of overexpression the LINC317.5 on transcription factors of HepG2-IR model with qRT-PCR. The housekeeping gene which was used to establish the relative expression of the analyzed genes was β-actin. **F** The lipid metabolism-related protein expression of overexpression the LINC317.5 in the HepG2-IR (ACADM, CPT1A, FAS, ACC1). Western blot experiments have been repeated three times (The relative expression was calculated based on the expression level in the O-Control group. The relative proliferation was calculated based on the proliferation level in the O-Control group. The relative apoptotic rate was calculated based on the apoptotic rate in the O-Control group. *P < 0.05, **P < 0.01, ***P < 0.001) (TG: triglyceride).

To investigate the effects of LINC317.5 on TG accumulation, HepG2-IR were treated with interference expression and overexpression. The content of intracellular TG in interference expression of LINC317.5 were markedly increased compared with control group (Fig. 3A), and in overexpression of LINC317.5 were markedly decreased compared with control group (Fig. 4A). Both of interference expression and overexpression of LINC317.5, there were no significant effect on the cell proliferation of HepG2-IR (Figs. 3B and 4B). However, the cell apoptosis of interference expression of LINC317.5 was markedly higher than the control group of HepG2-IR (Fig. 3C), and of overexpression of LINC317.5 was markedly lower than the control group of HepG2-IR (Fig. 4C). Also, the target mRNA *TKFC* was detected as gene level and protein level. Both of gene level and protein level, the *TKFC* was markedly higher in the interference expression of LINC317.5 than that in the control group in (Fig. 3D), and markedly lower in the overexpression of LINC317.5 than that in the control group (Fig. 4D).

To better understand the underlying mechanism of LINC317.5 on lipid metabolism in HepG2, we tested the related transcription factor (*PPARα, PPARγ, and SEPER-1c*) and the related protein expression (*ACADM, CPT1A, FAS*, and *ACC1*) (Supplemental Data 2). Compared with control group, the relative expression of *PPARα* and *PPARγ* were decreasing, and *SREBP-1c* was increasing in the LINC317.5-interfering group (Fig. 3E). Relatively, the relative expression of *PPARα* and *PPARγ* were increasing, and *SREBP-1c* was decreasing in the LINC317.5-overexpressing group (Fig. 4E). As shown in Fig. 3F, interference expression of LINC317.5 significantly decreased the protein expression of *ACADM* and *CPT1A*, whereas increased the protein expression of *FAS* and *ACC1*. As shown in Fig. 4F, overexpression of LINC317.5 significantly increased the protein expression of *ACADM*, whereas decreased the protein expression of *FAS* and *ACC1*, no significant difference was observed for the protein expression of *CPT1A*.

### Effects of LINC317.5 and mRNA-*TKFC* on TG accumulation in HepG2-IR

**Fig. 5:**
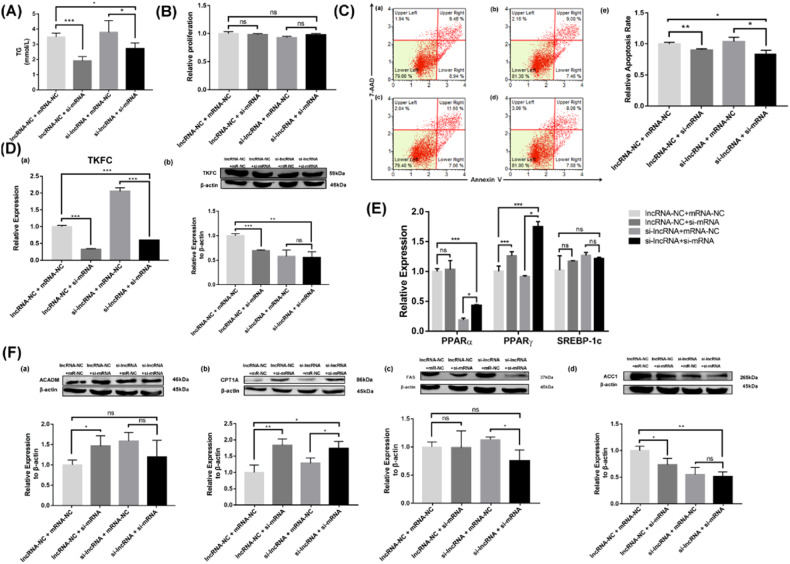
LINC317.5 binding to *TKFC* regulate the lipid metabolism-related gene, transcription factor, and protein expressions in the HepG2-IR model. **A** The TG level of knockdown of LINC317.5 binding to *TKFC* in the HepG2-IR model. **B** The effect of knockdown of LINC317.5 binding to *TKFC* on the activity of HepG2-IR models with CCK-8. **C** The effect of knockdown of LINC317.5 binding to *TKFC* on apoptosis of HepG2-IR model with flow cytometry (**a**) lncRNA-NC+mR-NC; (**b**) lncRNA-NC+si-mR; (**c**) si-lncRNA+mR-NC; (**d**) si-lncRNA+si-mR; (**e**) Relative apoptosis rate. **D** The *TKFC* relative expression after LINC317.5 binding to *TKFC* in the HepG2-IR model; (**a**) for gene expression by qRT-PCR and (**b**) for protein expression by western blot. **E** The effect of knockdown of LINC317.5 binding to *TKFC* on transcription factors of HepG2-IR model with qRT-PCR. The housekeeping gene which was used to establish the relative expression of the analyzed genes was β-actin. **F** The lipid metabolism-related protein expression of knockdown of LINC317.5 binding to *TKFC* in the HepG2-IR (ACADM, CPT1A, FAS, ACC1). Western blot experiments have been repeated three times (The relative expression was calculated based on the expression level in the lncRNA-NC + mRNA-NC group. The relative proliferation was calculated based on the proliferation level in the lncRNA-NC + mRNA-NC group. The relative apoptotic rate was calculated based on the apoptotic rate in the lncRNA-NC + mRNA-NC group. *P < 0.05, **P < 0.01, ***P < 0.001) (TG: triglyceride).

Since *TKFC* was identified as the target mRNA of LINC317.5, we conducted experiments to explore whether *TKFC* mitigates the impact of LINC317.5 on triglyceride (TG) accumulation. We interfered cells with both LINC317.5 and *TKFC*. Our results, depicted in Fig. 5A, revealed a significant decrease of TG levels in cells expressing *TKFC* compared to the control group, indicating that *TKFC* reduced TG accumulation induced by LINC317.5. However, there was no notable effect on the cell proliferation of HepG2-IR in any rescue group (Fig. 5B). Furthermore, the cell apoptosis rate in cells with reduced *TKFC* expression was notably lower than that in the control group of HepG2-IR, as well as in cells with reduced expression of both *TKFC* and LINC317.5 (Fig. 5C). The expression levels of *TKFC*, at both the gene and protein levels, were decreased after interfering with *TKFC* expression (Fig. 5D).

To better understand the underlying mechanism of both mRNA *TKFC* and LINC317.5 on lipid metabolism in HepG2, we examined the expression of related transcription factors (PPARα, PPARγ, and SEPER-1c) and proteins (ACADM, CPT1A, FAS, and ACC1). Compared to the lncRNA+si-mRNA group, the expression levels of PPARα and PPARγ were increased in the si-lncRNA+si-mRNA group. Following LINC317.5-intervention, the reduction in *TKFC* expression led to decreased PPARα-accumulation and reversed the expression of PPARγ. However, there was no significant different of *SEPER-1c* between every rescue group (Fig. 5E).

In addition, as shown in Fig. 5F, the interference with both *TKFC* mRNA and LINC317.5 significantly elevated the protein-expression of CPT1A while decreasing the protein-expression of ACC1, with no significant changes in the expression of ACADM and FAS compared to the control group.

## Discussion

Both from a public health perspective or a clinical perspective, it is necessary to pay more attention to abnormal glucose patients with HTG due to the combined effect. We aimed to identify the key lncRNA of HTG in the abnormal glucose metabolism patients, and validate the key lncRNA in a larger sample size of population and vitro experiments. The main findings are as following: Firstly, LINC317.5 down-regulated in the HTG with abnormal glucose patients. Secondly, LINC317.5 affect the accumulation of TG might through regulating the transcription factors (*PPARα, PPARγ*, and *SEPER-1c*) and related protein (*ACADM, CPT1A, FAS, ACC1*) expression of lipid metabolism. Thirdly, mRNA *TKFC* could partly affect the accumulation of TG of LINC317.5. Our results might provide the information of non-invasive biomarkers for the HTG with abnormal glucose.

LINC317.5 was cytochrome b561 family member A3 (CYB561A3) in the Ensembl database. CYB561A3, also called lysosomal Cyt561 or LCytb, is expressed in late endosomal/lysosomal membranes in macrophages [15]. Lipids are degraded in lysosomes, the LDL-derived cholesteryl ester and triglycerides are hydrolyzed to cholesterol and fatty acids by lysosomal acid lipase (LAL) [16, 17]. Autophagy is an important intracellular catabolic process that allows cell components such as damaged organelles and unfolded proteins to be degraded by lysosomes [18]. The previous study termed the interrelationship between autophagy and lipid metabolism as macrolipophagy, which is distinct from canonical autophagic mechanisms [19]. Direct lipid drops ingestion by lysosomes contributes in a meaningful way to lipid catabolism in the hepatocyte and occurs by a lysosome-directed process [19].

*TKFC* was the target mRNA of LINC317.5 in this study by bioinformatics analysis. It was indicated that might be involved in the occurrence and progression of Alcoholic fatty liver disease [20]. Moreover, *TKFC* deficiency could reduce hepatic triglyceride accumulation in multiple mouse models [21]. KEGG database showed that *TKFC* (2.7.1.29) could participate in the glycerolipid metabolism pathway (map00561) and indirectly affect triglyceride [14], which was consistent to our result of KEGG analysis.

It is well known that insulin resistance is a core defect in T2DM [22]. In 1995, Stern proposed the “common soil theory”, which indicated that the common soil was IR, in which these diseases would grow [23]. IR is regarded as a “silent” risk factor, and an important cause of T2DM or other metabolic diseases [24]. As a cell culture model retains the morphology and most of function in culture, the HepG2 is widely used for biochemical and nutritional studies, such as hepatic glucose production, the modulation of the insulin pathway and oxidative stress in vitro [25, 26]. In this study, HepG2-IR was used to validate the LINC317.5 in vitro experiments to explore the TG level and expression of related transcription factors and related enzyme of lipid metabolism.

*PPARα* (PPAR) is part of the nuclear receptor superfamily, which is highly expressed in liver [27]. PPAR is a master regulator of lipid metabolism, *PPARα* promotes fatty acid β-oxidation in the liver [28, 29]. *PPAR-γ* activates various genes directly involved in lipid storage/ release and cell differentiation of fats. Previously, it has been reported that overexpression of *PPARγ* leads to ectopic fat deposition in the liver [30]. Sterol regulatory element-binding protein (*SREBP1*) is a crucial transcription factor for the enzymes of de novo lipogenesis, mainly regulates the expression of key adipogenic genes such as *FAS* and *ACC1*, whose activities are under the control of activated protein kinase (*AMPK*) [20, 31]. The continuous activation of *SREBP1* triggers hepatic steatosis by enhancing TAG accumulation, which happens in high-calorie or high-fat diet populations [32].

The expression of acetyl-CoA carboxylase α (*ACCα*) and *FAS* rate-limiting enzymes involved in lipogenesis was upregulated with TG accumulation [33]. With fatty acid uptake, hepatic insulin resistance leads to impairment of glucose homeostasis, decreases fatty acid oxidation, and contributes to TG accumulation. In addition, the de novo lipogenesis pathway comprises glycolysis biosynthesis of saturated fatty acid followed by desaturation and TG formation. Key rate-limiting enzymes in this process include glucokinase and liver-type pyruvate kinase in glycolysis *ACC*, and *FAS* [34]. The liver can synthesize new fatty acids from acetyl-CoA by the action of *ACC* and *FAS*. Initially, *ACC* catalysis acetyl-CoA converts to malonyl-CoA and then to palmitate by *FAS* [34]. In NAFLD, the expression of *ACC* and *FAS* increase in response to the increase of upstream *SREBP-1* [35, 36]. Both of *ACADM* and *CPT1A* were explored the relationship with the lipid metabolism [37]. For fatty oxidation, *PPARα* is a key nuclear receptor that controls the rate of oxidation of fatty acids occurring in the mitochondria and is also related to *CPT1* [38].

Our study found that interfering of LINC317.5 would increase *SREBP-1C*, *FAS*, *ACC1*, *TKFC* and TG, decrease *PPARα*, *PPARγ*, *ACADM* and *CPT1A*. Meanwhile interfering of *TKFC* would decrease TG. When we added interfering of *TKFC* to the interfering of LINC317.5, the change trend of *PPARα*, *PPARγ*, *CPT1A*, *ACC1* and TG changed, and the significant change of *SREBP-1C*, *FAS* and *ACADM* disappeared. This indicated that LINC317.5 might affect TG level through *SREBP-1C*, *FAS*, *ACC1*, *PPARα*, *PPARγ*, *ACADM* and *CPT1A*, and this process would be affected by *TKFC*.

In conclusion, LINC317.5 would decrease in patients with HTG and abnormal glucose and probably affect the accumulation of TG through regulating the transcription factors (*PPARα, PPARγ*, and *SEPER-1c*) and related protein (*ACADM, CPT1A, FAS, ACC1*). *TKFC* could partly affect this process. Therefore, we suggested LINC317.5-*TKFC* as a novel biomarker for hypertriglyceridemia in the abnormal glucose metabolism. Our results might provide the information of non-invasive biomarkers for the HTG with abnormal glucose.

## Materials and methods

### Participants

24 Chinese patients with abnormal glucose metabolism participated in this study, 12 of them had HTG and 12 had normal triglyceride (NTG) (Supplemental Data 1). They were recruited at the First Hospital of Jilin University from July to September in 2020. Mean age of them was 53.5 years. All participants have written informed consent and the study was approved by the Ethics Committee of the Public Health of the Jilin University, and the privacy of the participants are strictly confidential.

The diagnostic criteria of abnormal glucose metabolism and HTG were based on the “Guidelines for the Prevention and Control of Type 2 Diabetes in China” (2017 Edition) and the “Guidelines for Prevention and Treatment of Dyslipidemia in Adults in China” (2016 Edition). Abnormal glucose metabolism was defined as fasting plasma glucose (FPG) ≥ 6.1 mmol/L or oral glucose tolerance test (OGTT) 2-h blood glucose ≥ 7.8 mmol/L. HTG was defined as TG ≥ 1.7 mmol/L. Patients who have used drugs or other treatments to control blood glucose or TG in the past, or have a history of coronary artery disease (CAD), hypertension, atrial fibrillation, myocardial infarction, tumor, acute infectious disease, immune disease or hematological disease were excluded from this study.

### Blood sample collection and RNA sequencing

Once we collected the blood samples, Trizol (TAKARA BIO INC., CA, Japan) was added immediately. We used total RNA extraction kit to isolate and purify total RNAs. Then we test the purity of RNAs by NanoPhotometer® spectrophotometer (IMPLEN, CA, USA). We used RNA Nano 6000 Assay Kit of the Agilent Bioanalyzer 2100 system (Agilent Technologies, CA, USA) to evaluate the integrity of RNAs.

The chain-specific library was constructed with ribosomal RNA removing, and was sequenced according to pooling of the effective concentration of the library and the data output requirements, which using the Illumina PE150. The reads with adapter and N (N means that the nucleobase information cannot be determined) ≥ 0.002 and with low-quality from raw data were removed for followed sequencing with calculating Q20, Q30, and GC content additionally. We used the clean data obtained through the above criteria to conduct all the analyses in this study.

### Construction of WGCNA

The “WGCNA” [39] package in R-Studio 4.0.4 software is a comprehensive collection of R functions for performing various aspects of weighted correlation network analysis [40]. We used it to analyze data. WGCNA analysis focuses on the association between the sample trait and a few modules, instead of describing the correlation structure between thousands of genes and a sample trait [13]. In the WGCNA algorithm, the elements in the co‑expression matrix of the genes were not the correlation coefficients of the genes, but rather the weighted value of the correlation coefficients [41]. We selected the lncRNAs with top 5000 median absolute deviation (MAD) for the subsequent analysis to ensure the heterogeneity and accuracy of bioinformatics for co‐expression network analysis. We calculated pearson correlation coefficient for all the genes, then selected an appropriate soft threshold *β* (0.85) automatically by the pickSoft-Threshold function of the WGCNA package whose function was to amplify the correlation between genes [42]. Subsequently, we transformed the adjacency matrix into topological overlap matrix (TOM), used it to describe the similarity of gene expression, and used 1-Tom to represent the heterogeneity between genes.

Finally, we used dynamic tree to divide the modules of hierarchical clustering results, and merge the modules with lncRNAs < 30 and cutting height < 0.25 [43].

### Screening for key modules and identification of key lncRNAs and functional enrichment analysis

Based on the above analysis, we subdivided five thousand genes into several modules. Module referred to a set of genes in which the expression mode highly correlated with the sample and the first principal component module characteristic genes (MEs) were calculated to express the expression level of the gene module. If the *P*-value < 0.05, the module was believed to be correlated with HTG in abnormal glucose metabolism.

We take the intersections of genes in correlated module and differentially expressed genes by RNA sequencing into the representative Kyoto Encyclopedia of Genes and Genomes (KEGG) pathways analysis for further elucidation of the functional properties.

### Validation in the GEO data set

GSE130991 was a previously published GEO data. Its data and sample collection took place in France between 2006 and 2016 [44]. In GSE130991, 98 patients with abnormal glucose metabolism met our criteria, 34 of them with HTG and 64 of them with normal TG level. We obtained data of GSE130991 by GPL20265 (HTA-2_0) Affymetrix Human Transcriptome Array 2.0. We analyzed the data in Partek Genomics Suite 6.6, normalized them using RMA, and log2 transformed them. We also performed differential expression analyses of genes in GSE130991 using a *t*-test based on these data. Then we obtained individual p-values and log2 values (fold change). Finally, we succeeded in using GSE130991 to observe the expression levels of the selected lncRNAs in the two groups.

### Quantitative real‐time polymerase chain reaction (qRT‐PCR)

We used G*Power 3.1.9.7 software for sample size estimation, the parameters were set as follows: effect size of 0.5, *α* = 0.05, 1-β = 0.95, two-tailed test, and equal sample sizes for both groups. After calculation, it was determined that each group should have 70 samples. To ensure adequate sample size, the final decision was made to increase the sample size to 80 per group.

We collected blood samples for qRT-PCR experiment at the First Hospital of Jilin University from July 5th to 19th 2021. There were 164 patients with abnormal glucose metabolism, including 82 patients with HTG and 82 with NTG, respectively. We used MolPure® Blood RNA Kit (19241ES50, YEASEN) to extract total RNA based on the manufacturer’s instructions. Subsequently, we used lnRcute lncRNA First-Strand cDNA Kit (KR202, TIANGEN) to conduct reverse transcription. The cDNA was then analyzed by qRT-PCR using lnRcute lncRNA qPCR Kit (FP402, TIANGEN) on QuantStudio 3 system (Applied Biosystems). Besides, we used pearson correlation analysis to determine the correlation between the relative expressions of lncRNAs and TG, setting the significance as *P* < 0.05.

Moreover, we have examined the relative expression of target mRNA-*TKFC* and lipid mentalism related transcription factor (*PPARα, PPARγ, and SEPER-1c*) using M5 Sprint qPCR RT kit with gDNA remover and 2 × M5 HiPer SYBR Premix EsTaq (Mei5Bio; Beijing, China). Expression data were normalized to the expression of β-actin with the 2 − ΔΔCt method. Table 1 lists the sequences of the primers used in this study.

**Table 1:** Sequence of primers for quantitative real-time PCR.

RNA	primers
LINC317.5	F: 5′- TTCCTTGCTGAGACCCACATTGC -3′
	R: 5′- TTCCCGCTCTCCACCCTATTTCC -3′
*TKFC*	F: 5’-GTGGAGATGGTGGTGATTG-3’
	R: 5’-CCTGCCACCTTGTGTATAA-3’
PPARα	F: 5’- CTCTGGCAGCGAATGTAGGAAGTC-3’
	R: 5’- GCACGGTAGACCAAGGCTGTTAG-3’
PPARγ	F: 5’ GCCCTTCACTACTGTTGACTTCTCC-3’
	R: 5’- CAGGCTCCACTTTGATTGCACTTTG-3’
SEPER-1c	F: 5’- GACTGACTTCCAGGACCTGTTGTG-3’
	R: 5’- GGAGGAGGCTTCTTTGCTGTGAG-3’
HS-ACTB	F: 5’-CCTGGCACCCAGCACAAT -3'
	R: 5’-GGGCCGGACTCGTCATAC -3'

lncRNA, long noncoding RNA; PPARα, peroxisome proliferator-activated receptor alpha; PPARγ, peroxisome proliferator-activated receptor gamma; SREBP-1c, sterol regulatory element-binding protein-1c; LINC317.5, ENST00000540317.5.

### Fluorescence in situ hybridization (FISH)

We observed the distribution of lncRNA in the HepG2 by fluorescence in situ hybridization (FISH) Kit (Ribo^TM^ lncRNA FISH Probe Mix and Ribo^TM^ Fluorescent In Situ Hybridization; RiboBio, China). We used RiboBio to design and synthesize Cy3-labeled LINC317.5 probes, U6 probes, and 18 S probes. Both U6 and 18 S were the internal reference, U6 was almost all located in the nucleus while 18 S was almost all located in the cytoplasm.

### Cell culture of insulin resistant-HepG2 (HepG2-IR) cells and biochemical detection

According to a previously described method with minor modifications [45, 46], we induced IR in HepG2 cells. Insulin resistance cell model was established with insulin (I8040-Insulin from bovine pancreas, Solarbio; Beijing, China) at a concentration of 0, 0.1, 1, 10 μmol/L. After inducing for 24 h or 48 h, determine the normalized extracellular glucose content in the culture supernatant and TG produced by HepG2 cell, to definite the induced condition of HepG2-IR. We estimated the concentration of glucose and TG using commercial kits (Nanjing Jiancheng Bioengineering Institute; Nanjing, China).

### SiRNA and plasmid construction and transfection

The vectors si-LINC317.5, si-mRNA-*TKFC*, and si-NC are chemically modified small RNAs, we constructed them by GenePharma (Suzhou, China). We applied Vector si-lncRNA and si-mRNA to inhibit endogenous si-LINC317.5 and si-mRNA-*TKFC* activity by silencing LINC317.5 and mRNA *TKFC*. We purchased LINC317.5-overexpressing and overexpression-control lentiviruses from Public Protein/Plasmid Library (China), and we used the O-LINC317.5 lentivirus to upregulate LINC317.5 activity. We used SiRNA at 10 nmol/L, O-lncRNA at 2.5 μg/L for transfection with lipofectamine 2000 (Invitrogen, USA), and the same amount of si-NC and O-Control for transfection. (Supplemental Data 3).

### Cell proliferation assay by CCK-8

We conducted Cell Counting Kit-8 (CCK-8) to test cell viability. We put cells into 96-well plates at l × l0^4^/well in complete medium and cultured them for 24 h. After related treatment with transfection and seting up HepG2-IR, 10 μL of CCK-8 reagent was inserted to each well, and the plates were further incubated for 1.5 h. We measured the spectrophotometric absorbance at 450 nm of each sample.

### Cell apoptosis assays by flow cytometry

For apoptosis analysis, we put cells in 6-well plates at the destiny of approximate 5 × l0^4^/well. After related treatment with transfection and setting up HepG2-IR, we washed them twice with cold PBS and re-suspended them in binding buffer with a density of 1 × 10^6^/mL. Cells were next stained with AnnexinV-PE (BD Biosciences) for 15 min, the signal was acquired and analyzed by Guava Muse Cell Analyzer (Luminex, USA).

### Western blot assay

Cell proteins were extracted and separated by 10% SDS-PAGE gels and transferred to 0.22 μm NC membranes (Millipore, USA). The membranes were blocked with 5% skim milk powder and incubated with specific antibodies at 4 °C overnight. Then we incubated the membranes with the appropriate secondary antibodies, and used an ECL detection system (Solarbio; Beijing, China) to detect the protein bands. We used β-actin (13E5; Cell Signaling Technology) as a control. The primary antibodies are included: *ACADM* (DF6670; Affinity)*, CPT1A* (DF12004; Affinity)*, FAS* (AF5342; Affinity)*, ACC1* (AF6421; Affinity), and *TKFC* (AF0661; Affinity). The secondary antibody was IgG H&L (HRP) (ab205718; Abcam). The dilution used for the antibodies was Antibody Dilution Buffer (Solarbio, A1800).

### Statistical analysis

We used GraphPad Prism 7.0 software (San Diego, CA, USA) to perform statistical analysis. All experiments were repeated at least three times in parallel, and data were presented as mean ± SD. To analyze statistical significance between the two groups, we used two-tailed Student’s *t*-test. We performed one-way analysis of variance (ANOVA) for multiple groups. Pearson correlation analysis was employed to identify the correlation between variables. *P* < 0.05 was assumed as statistically significant.

### Supplementary information

Supplemental Data 1

Supplemental Data 2

Supplemental Data 3

## Data Availability

Our data have not been shared openly at present to protect participants’ privacy.

## Code availability

The authors declare that there is no custom code was used.
