# Publisher Correction: LINC317.5 as a novel biomarker for hypertriglyceridemia in abnormal glucose metabolism

**DOI:** 10.1038/s41420-024-02081-5

**Published:** 2024-07-03

**Authors:** Yixue Yang, Mengzi Sun, Shoumeng Yan, Nan Yao, Xiaotong Li, Caihong Wu, Zibo Wu, Fengdan Wang, Weiwei Cui, Bo Li

**Affiliations:** 1 Department of Epidemiology and Biostatistics, School of Public Health, Jilin University, Changchun, 130021 P. R. China; 2 The First Affiliated Hospital of Xi’an Jiaotong University, International Obesity and Metabolic Disease Research Center, Xi’an, 710061 P. R. China; 3 Global Health Institute, Xi’an Jiaotong University, Xi’an, 710115 P. R. China; 4 School of Nursing, Jilin University, Changchun, 130021 P. R. China; 5 Department of Nutrition and Food Hygiene, School of Public Health, Jilin University, Changchun, 130021 P. R. China

**Keywords:** Gene regulation, Long non-coding RNAs, Dyslipidaemias

Correction to: *Cell Death Discovery* 10.1038/s41420-024-01968-7, published online 26 April 2024

In this article the article title has been given erroneously:

“LINC317.5 as a Novel Biomarker for Hypertriglyceridemia in Normal Glucose Metabolism”

It should read:

“LINC317.5 as a Novel Biomarker for Hypertriglyceridemia in Abnormal Glucose Metabolism”

The original article has been updated.

