# Supplementary material for: LINC317.5 as a novel biomarker for hypertriglyceridemia in abnormal glucose metabolism
Source: Cell Death Discov. 2024 Apr 26;10:194. doi: 10.1038/s41420-024-01968-7 (PMC11053116; doi:10.1038/s41420-024-01968-7)
Supplement: Supplementary file 1 — Supplemental Data 1 [file 41420_2024_1968_MOESM1_ESM.docx]

**Supplemental Data 1. Category Matching of Sequencing Samples**

1. **Normality Test**

| **Index** | **z of HTG Group** | **p of HTG Group** | **z of Control Group** | **p of Control Group** |
| --- | --- | --- | --- | --- |
| Age | 0.963 | 0.825 | 0.906 | 0.191 |
| Height (cm) | 0.853 | 0.040 | 0.904 | 0.178 |
| Weight (kg) | 0.863 | 0.053 | 0.821 | 0.017 |
| BMI | 0.944 | 0.546 | 0.771 | 0.004 |
| FBG (mmol/L) | 0.753 | 0.003 | 0.759 | 0.003 |

1. **Category Matching**

| **Index** | | **HTG Group** | **Control Group** | t/z/χ^2^ | P |
| --- | --- | --- | --- | --- | --- |
| Gender | 1: male | 8 (66.67%) | 6 (50%) | 0.686 | 0.408 |
|  | 2: female | 4 (33.33%) | 6 (50%) |  |  |
| Age | | 53.58±4.60 | 53.42±7.99 | 0.063 | 0.951 |
| Height (cm) | | 170.00 (168.25, 174.25) | 168.00 (163.50, 171.50) | -1.279 | 0.201 |
| Weight (kg) | | 65.00 (63.50, 68.75) | 65.00 (63.50, 67.25) | -0.031 | 0.975 |
| BMI | | 21.97 (21.22, 24.22) | 23.01 (21.87, 24.40) | -0.752 | 0.452 |
| FBG (mmol/L) | | 7.00 (6.53, 8.80) | 8.25 (6.60, 12.30) | -0.815 | 0.415 |

Normally distributed continuous variables were represented using mean ± standard deviation, non-normally distributed continuous variables were represented using median (P25, P75), categorical variables were represented using frequency (percentage).

Inter-group difference analysis: Independent sample t-test was used for normally distributed continuous variables, Mann-Whitney U test was used for non-normally distributed continuous variables, and chi-square test was used for categorical variables.
