# Supplementary material for: LINC317.5 as a novel biomarker for hypertriglyceridemia in abnormal glucose metabolism
Source: Cell Death Discov. 2024 Apr 26;10:194. doi: 10.1038/s41420-024-01968-7 (PMC11053116; doi:10.1038/s41420-024-01968-7)

Uncropped Western Blot Figures

1.uncropped figure of Figure 3(D)(b)

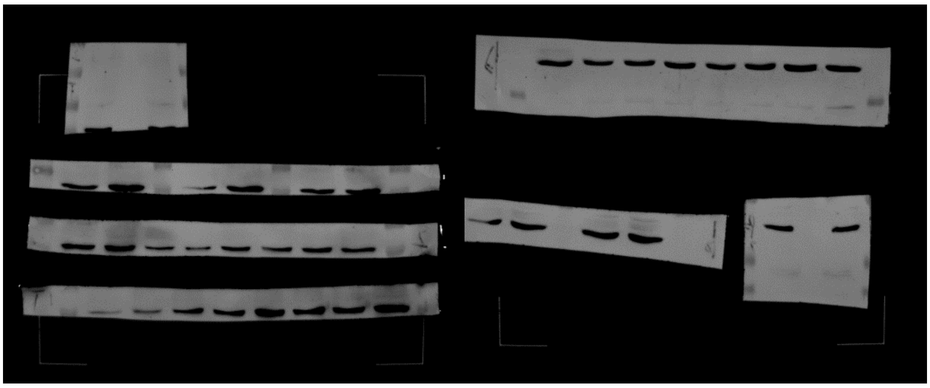

2.uncropped figure of Figure 3(F)(a)

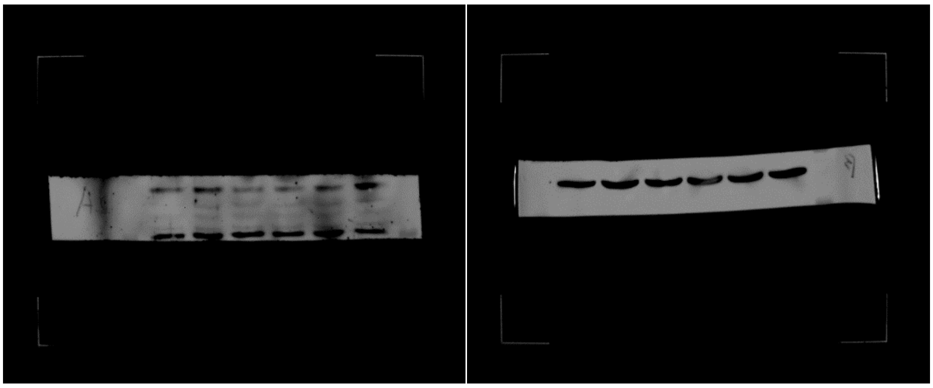

3.uncropped figure of Figure 3(F)(b)

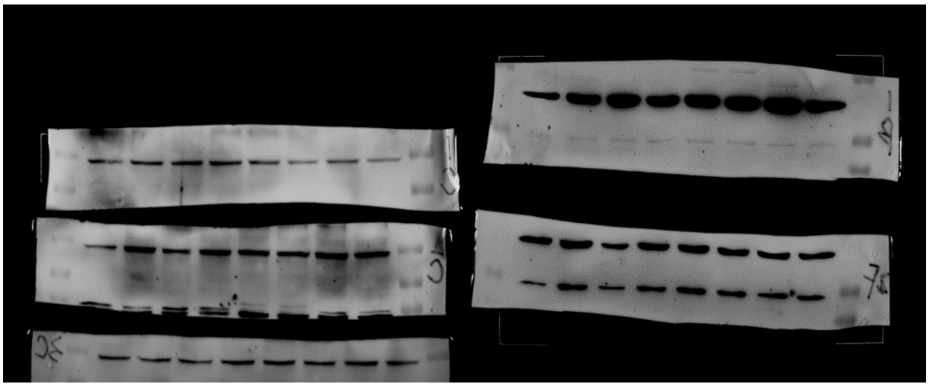

4.uncropped figure of Figure 3(F)(c)

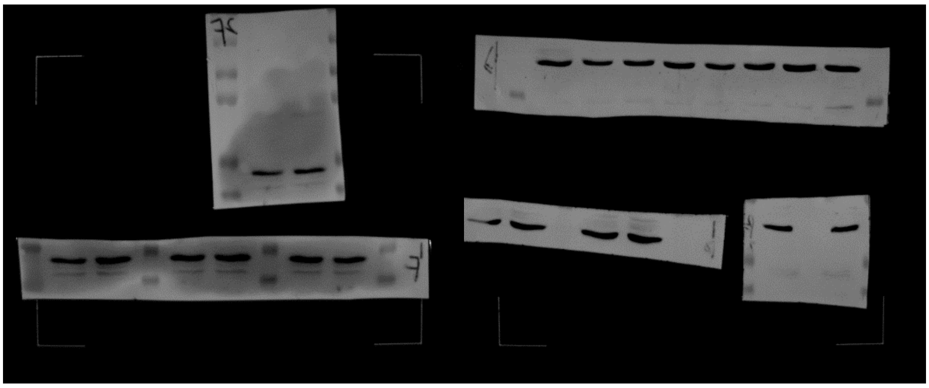

5.uncropped figure of Figure 3(F)(d)

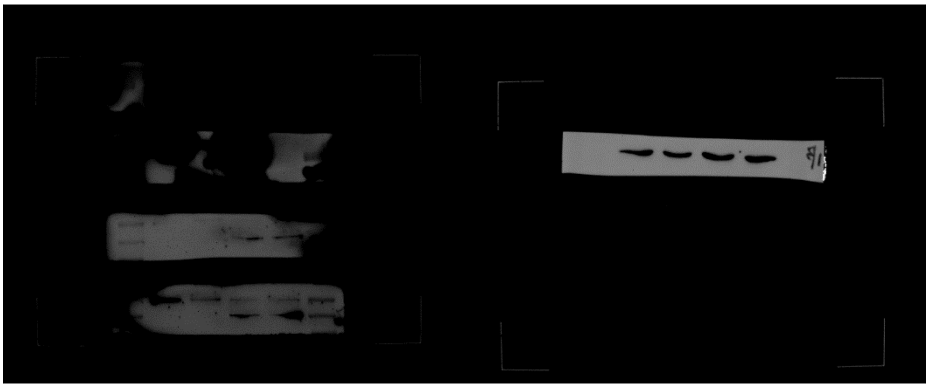

6.uncropped figure of Figure 4(D)(b)

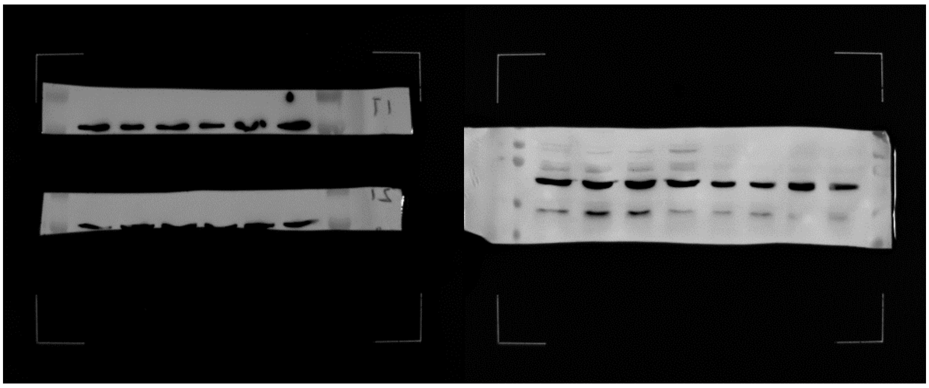

7.uncropped figure of Figure 4(F)(a)

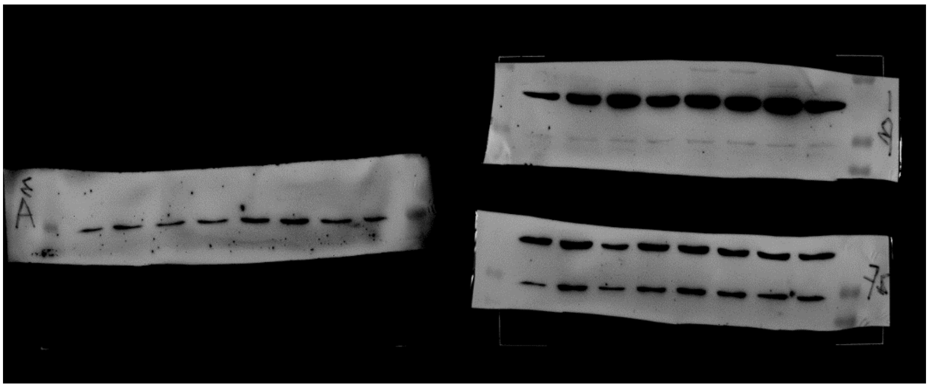

8.uncropped figure of Figure 4(F)(b)

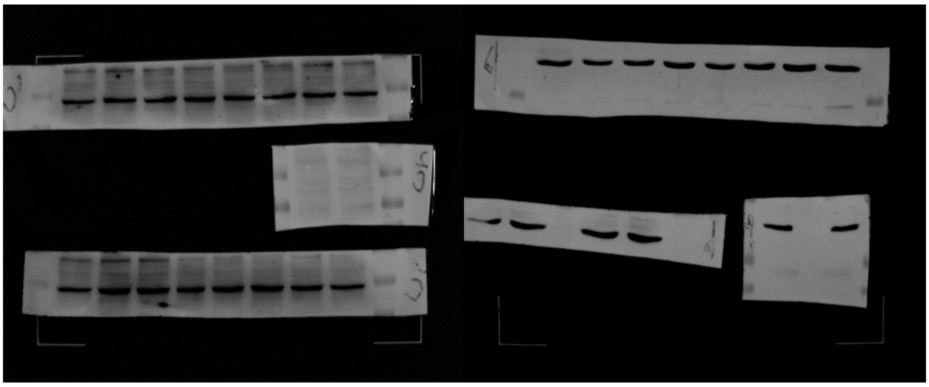

9.uncropped figure of Figure 4(F)(c)

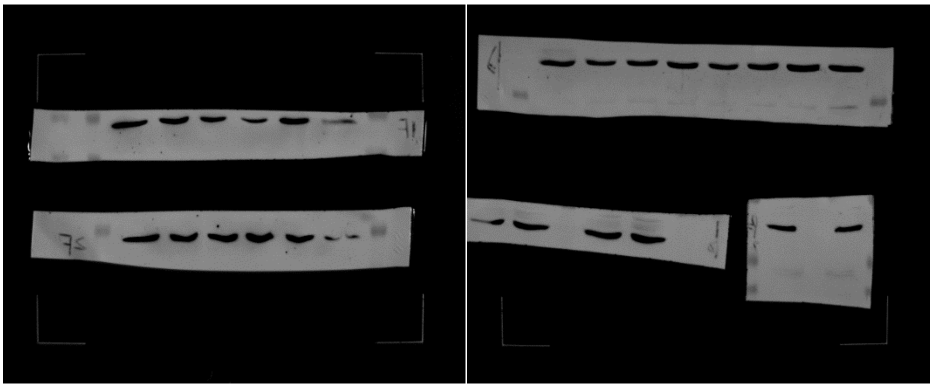

10.uncropped figure of Figure 4(F)(d)

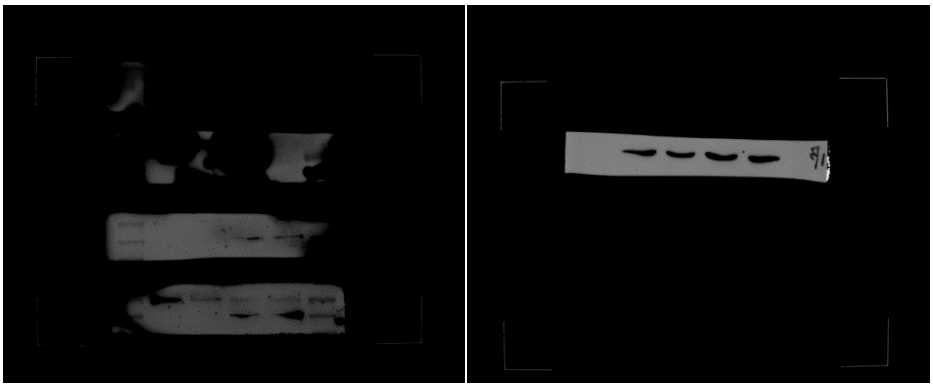

11.uncropped figure of Figure 5(D)(b)

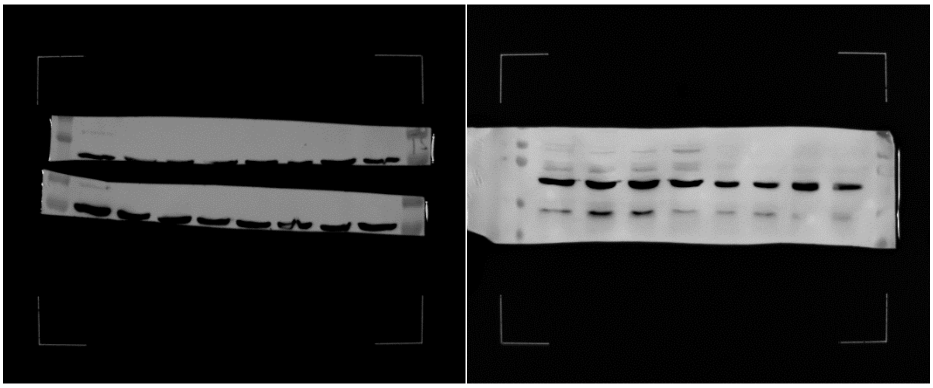

12. uncropped figure of Figure 5(F)(a)

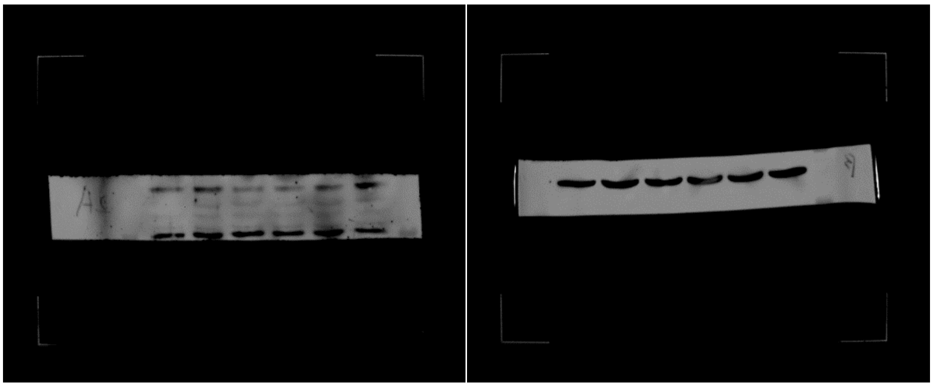

13.uncropped figure of Figure 5(F)(b)

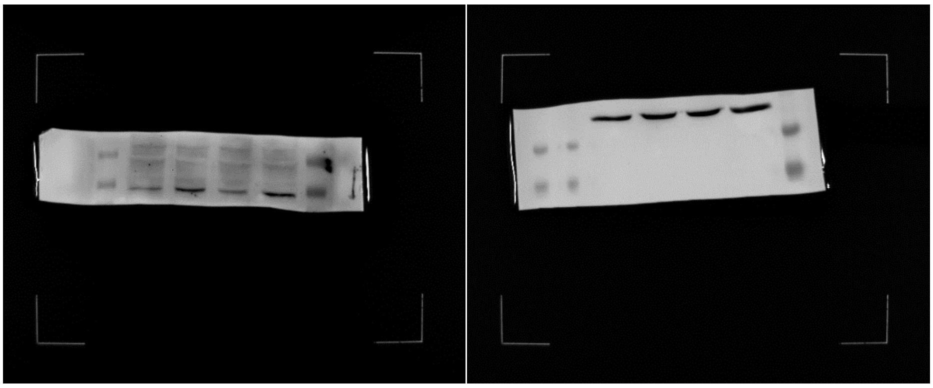

14.uncropped figure of Figure 5(F)(c)

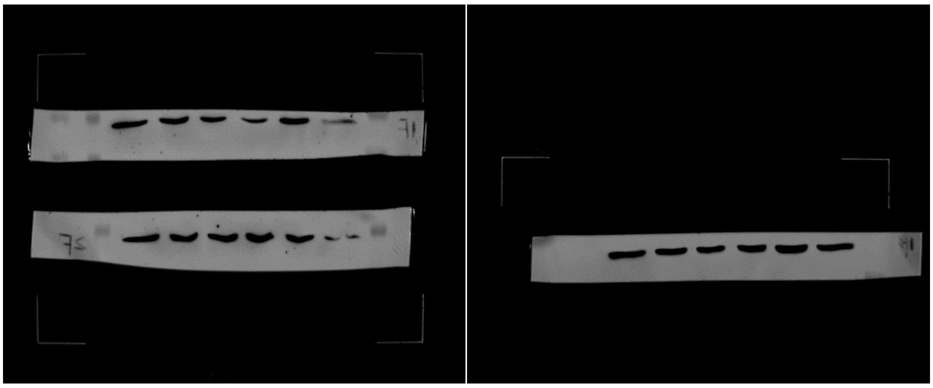

15.uncropped figure of Figure 5(F)(d)

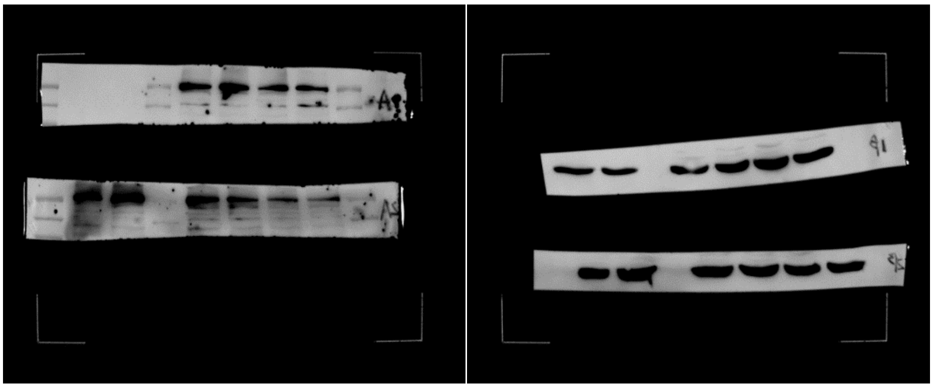

Supplement: Supplementary file 2 — Supplemental Data 2 [file 41420_2024_1968_MOESM2_ESM.pdf]
