# Supplementary material for: LINC317.5 as a novel biomarker for hypertriglyceridemia in abnormal glucose metabolism
Source: Cell Death Discov. 2024 Apr 26;10:194. doi: 10.1038/s41420-024-01968-7 (PMC11053116; doi:10.1038/s41420-024-01968-7)
Supplement: Supplementary file 3 — Supplemental Data 3 [file 41420_2024_1968_MOESM3_ESM.docx]

We used cationic liposome transfection reagent to transfect si-lncRNA for silencing the target lncRNA. After transfection for 24 hours, we extracted cellular RNA, then conducted reverse transcription to synthesize cDNA. RT-qPCR was performed to calculate relative expression levels of the target lncRNA.

**Design of si-lncRNA sequences**

| siRNA | sequences of primer |
| --- | --- |
| si-LNC317.5 | sense: 5’-GCUGAGACCCACAUUGCUUTT-3’  antisense: 5’-AAGCAAUGUGGGUCUCAGCTT-3’ |
| NC | sense: 5’-UUCUCCGAACGUGUCACGUTT-3’  antisense: 5’-ACGUGACACGUUCGGAGAATT-3’ |
